# Ternary Complexes of *cis*-(NH_3_)_2_PtCl_2_ (*cis*-DDP) With Guanosine (guo), Cytidine (cyd) and the Aminoacids Glycine (gly), L-Alanine (ala), L-2-Aminobutyric Acid (2-aba), L-Norvaline (nval) and L-Norleucine (nleu)

**DOI:** 10.1155/MBD.1997.57

**Published:** 1997

**Authors:** Erasmia Katsarou, Costas Charalambopoulos, Nick Hadjiliadis

**Affiliations:** 1 Laboratory of Inorganic and General Chemistry University of Ioannina Ioannina 451 10 Greece; 2 Medical Faculty Laboratory of Experimental Physiology University of Ioannina Ioannina 451 10 Greece

## Abstract

The ternary complexes of formulae *cis*-[(NH_3_)_2_Pt(nucl)(amac)]NO_3_, where nucl = guo and cyd (guanosine and cytidine) and amac = the deprotonated aminoacids glycine (gly), L-alanine (ala), L-2-aminobutyric acid (2-aba), L-norvaline (nval) and L-norleucine (nleu), were prepared from the reactions of the binary chelated ones *cis*-[(NH_3_)_2_Pt(nucl)(amac)]NO_3_ with the nucleosides.

They were characterized by ^1^H, ^13^C and ^195^Pt NMR and IR spectra, together with elemental analysis and conductivity measurements. The aminoacids coordinate with Pt(II) in the ternary complexes with their terminal -NH_2_ groups, guo through N_7_ and cyd through N_3_. Ligand-ligand hydrophobic interactions were also observed in the ternary complexes and were stronger with longer aliphatic chains of the aminoacids. The ^3^E sugar conformation increased by 5-7% in the ternary systems, as compared to the free nucleosides, while the percentage of the *gg* conformation remained almost constant and the one of the *anti* conformation of the sugar increased also slightly. Finally, the *h* conformer around the C_α_-C_β_ bonds of the aminoacids reached a maximum in the binary systems and decreased again considerably in the ternary ones.


### TERNARY COMPLEXES OF *cis*-$(NH_3)_2PtCl_2$ (*cis*-DDP) WITH GUANOSINE (guo), CYTIDINE (cyd) AND THE AMINOACIDS GLYCINE (gly), L-ALANINE (ala), L-2-AMINOBUTYRIC ACID (2-aba), L-NORVALINE (nval) AND L-NORLEUCINE (nleu)

Erasmia Katsarou[1], Costas Charalambopoulos[2] and Nick Hadjiliadis[1]*

[1] Laboratory of Inorganic and General Chemistry [2] Medical Faculty, Laboratory of Experimental Physiology
University of Ioannina, Ioannina 451 10, Greece

### Abstract

The ternary complexes of formulae *cis*-$[(NH_3)_2Pt(nucl)(amac)]NO_3$, where nucl=guo and cyd (guanosine and cytidine) and amac=the deprotonated aminoacids glycine (gly), L-alanine (ala), L-2-aminobutyric acid (2-aba), L-norvaline (nval) and L-norleucine (nleu), were prepared from the reactions of the binary chelated ones *cis*-$[(NH_3)_2Pt(amac)]NO_3$ with the nucleosides.

They were characterized by $^1H$, $^{13}C$ and $^{195}Pt$ NMR and IR spectra, together with elemental analysis and conductivity measurements. The aminoacids coordinate with Pt(II) in the ternary complexes with their terminal $-NH_2$ groups, guo through $N_7$ and cyd through $N_3$. Ligand-ligand hydrophobic interactions were also observed in the ternary complexes and were stronger with longer aliphatic chains of the aminoacids. The $^3E$ sugar conformation increased by 5-7% in the ternary systems, as compared to the free nucleosides, while the percentage of the *gg* conformation remained almost constant and the one of the *anti* conformation of the sugar increased also slightly. Finally, the *h* conformer around the $C_\alpha$-$C_\beta$ bonds of the aminoacids reached a maximum in the binary systems and decreased again considerably in the ternary ones.

### Introduction

In recent years, we have been studying ternary systems of Pt(II) and Pd(II) with nucleobases-nucleosides and aminoacids-peptides as the simpler models of DNA-Pt-protein crosslinks, known to take place with the anticancer drug *cis*-DDP and its inactive congener *trans*-DDP [1-11].

A large variety of complexes of general formulae, *cis*-$[(ino)_2Pt(amac)]Cl_2$, *cis*-$[(ino_2)Pt(amacH)Cl]Cl$, *trans*-$[(guo)_2Pt(amacH)_2]Cl_2$, *cis*-$[(NH_3)_2Pt(Nb)(amac)]NO_3$, *trans*-, *cis*-$[(NH_3)_2Pt(9\text{-}MeG)(dipeptide)](NO_3)_2$, *cis*-$[(guo)_2Pd(amac)]Cl$, *cis*-$[(guo)_2Pd(amacH)Cl]Cl$, *trans*-$[(nucl)_2Pd(dipeptide)_2]Cl_2$ and *cis*-$[(nucl)_2Pt(dipeptide\ esters)_2]Cl_2$ (ino=inosine, guo=guanosine, Nb=9-methylguanine, 1-methylcytosine, 9-MeG=9-methylguanine, nucl=guo, ino, cyd (cytidine), amacH=zwitterionic form of aminoacids).

Sugar conformations and ligand-ligand interactions of the bases and aminoacids-peptides, simultaneously coordinated with Pt(II) were examined in these studies. For example, the hydrophobic ligand-ligand interactions are usually stronger with the increase of the aliphatic side chain of the aminoacids-peptides, but are usually weaker with increasing distance from the bonding sites. They are stronger in the *cis*- rather than the *trans*-series of complexes [3,7,8,10]. Also, the *anti* conformation of the sugar was increasing in the ternary complexes, compared to the free ligands and it was larger in the *trans*- than the *cis*-ternary complexes.

The present paper concludes our studies on such simple ternary systems. It reports on complexes of the general formulae *cis*-$[(NH_3)_2Pt(nucl)(amac)]NO_3$, where nucl is guo or cyd (Structure I) and amac the conjugated bases of the aminoacids glycine, L-alanine, L-2-aminobutyric acid, L-norvaline and L-norleucine. Similar ligand-ligand interactions and variations in sugar conformation were also found here and are compared with the previous results.

### Experimental

### *Materials*

All the L-aminoacids together with guanosine and cytidine were purchased from Sigma Chemical Company and were used without further purification. *Cis*-DDP was prepared from $K_2PtCl_4$ according to published methods [12,13].

Structure I

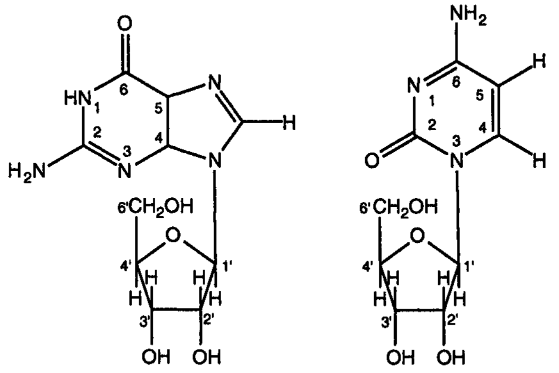

guanosine (guo) cytidine (cyd)

*Methods*

The elemental analysis (C,H,N) of the compounds was carried out on an EA-1108 Carlo Erba analyser at the Department of Chemistry of the University of Ioannina. The conductivity measurements were performed in an E365B Conductoscope, Metrohm Ltd., Herisau, Switzerland. The IR spectra were recorded in KBr pellets or nujol mulls on a Perkin-Elmer model 783 spectrophotometer, covering the region 4000-200 $cm^{-1}$. The $^1H$-NMR spectra were recorded at room or higher temperatures on a Brucker AMX-400 MHz or a DRX-600 MHz spectrometer in $D_2O$, with TSP as internal reference. The usual $^1H$ spectrometer conditions consisted of 6024 Hz and 6009 Hz sweep widths in the 400 MHz and 600 MHz instruments respectively. 16 scans and 16K data points were used. A line broadening factor of 0.3 Hz was used in processing the data. $^{13}C$ NMR spectra were recorded on the same spectrometer (Brucker AMX-400 MHz) at 100.62 MHz. A sweep width of 22727 Hz , 2500-3000 scans (sample concentration 20 mM) and 16K data points were used, as well as a line broadening factor of 2.5 Hz in processing the data.

$^{195}Pt$ NMR spectra were recorded on the Brucker AMX-400 MHz spectrometer at 86.02 MHz. Shifts are reported relative to $K_2PtCl_6$ (external standard 1630 ppm for $K_2PtCl_6$). Spectra were run typically with 40000 transients (sample concentration 20 mM) and a spectral width of 125 KHz. A pulse duration of 35.6 ms, followed by aquisition time of 65 ms and a delay time of 0.1 s was used. A line broadening factor of 25 Hz was used in the processing the data.

*Preparation of the Complexes*

*The binary complexes cis-$[(NH_3)_2Pt(amac)]NO_3$ where amac=glycine, L-alanine, L-2-aminobutyric acid, L-norvaline and L-norleucine.*

These were prepared according to previously described methods [14,15].

*The complexes cis-$[(NH_3)_2Pt(guo)(amac)]NO_3$, where amac are the anions of the above aminoacids and nucl are guanosine or cytidine.*

1.5 mmoles of guo or cyd were dissolved in about 100 ml of water under stirring at 60°C. 1 mmole of the complex *cis*-$[(NH_3)_2Pt(amac)]NO_3$ was added to the solution and this was stirred for 48 h at 60°C, in the dark. The pH of the solution was kept at 5 during the reaction, by addition of a $10^{-1}$ M solution of $HNO_3$. After cooling to 0°C and filtration of the unreacted guo, the solution was concentrated to 1 ml and passed through a Sephadex column (G-10, Pharmacia, 60 x 1.5 cm) using water as eluent. Fractions of 2 ml were collected. The ternary complexes of guo were eluted after unreacted binary ones (*cis*-$[(NH_3)_2Pt(amac)]NO_3$). Ocassionally, a second elution was required, in order to get pure complexes in larger amounts. The yields were varying 15-40 %.

*Deuterated Complexes*

These were prepared by dissolving the complexes in $D_2O$, followed by lyophilization.

**Results and Discussion**

The complexes were prepared according to the general reactions:

***cis*-$[(NH_3)_2Pt(H_2O)_2](NO_3)_2$+amacH→*cis*-$[(NH_3)_2Pt(amac)]NO_3$+$HNO_3$+$2H_2O$ (1)**

***cis*-$[(NH_3)_2Pt(amac)]NO_3$+ nucl → *cis*-$[(NH_3)_2Pt(amac)(nucl)]NO_3$ (2)**

(amacH=glycine, L-alanine, L-2-aminobutyric acid, L-norvaline, L-norleucine and nucl=guanosine, cytidine).

The elemental analyses are in agreement with the general formulae of the complexes. The molar conductance values confirm the 1:1 electrolytic nature of the complexes.

*Binding Sites*

$^1$H-NMR

The chemical shifts of all compounds are included in Table I. The $H_8$ proton of guo in the ternary complexes shifts downfield by about 0.6 ppm compared to the free ligand, while the $H_6$ and $H_5$ aromatic protons of cyd by only 0.07-0.10 ppm, indicating $N_7$ and $N_3$ coordination with the metal, of guo and cyd respectively [11,16-19]. These shifts are smaller than the ones caused by *cis*- and *trans*-DDP bound to the same nucleosides of about 1 ppm [17,19]. It was explained by the hydrophobic ligand-ligand interactions, taking place between the aminoacids and the nucleosides, bound simultaneously to the same metal [7,8,11].

$^{13}$C-NMR

The results obtained from the $^1$H-NMR spectra on the binding sites are confirmed by the $^{13}$C-NMR spectra of the compounds [20,21]. All $^{13}$C-NMR chemical shifts of the free aminoacids, the binary and the ternary complexes are given in Table II.

In the ternary complexes with guo all nucleoside carbon atoms are shifted downfield compared to the free ligand, with the most downfield shifted the $C_8$ atom (~7 ppm), near the $N_7$ binding site with Pt(II) of guo [20-22]. (See Table 2). In the cyd ternary complexes on the other hand, the $C_2$ atom near the binding site is shifted upfield by ~3 ppm, thus confirming the $N_3$ coordination with Pt(II) in solution [23,24]. The other base carbon atoms are not considerably shifted on passing from free cyd to its ternary complexes. It is worthwhile mentioning that most of cyd carbon atoms including the sugar are observed as doublets in the ternary complexes (Table II), thus confirming the hindered rotation around the Pt-$N_3$ bond, observed also in the $^1$H-NMR spectra [24].

The aminoacid carbon atoms on the other hand, are also not considerably shifted in the complexes, except the $C_a$ atoms near the -$NH_2$ coordination site with Pt(II) (2-3 ppm on passing from the free aminoacids to the binary complexes and ~6 ppm to the ternary ones) [11].

Finally, the terminal carboxylate carbons of the aminoacids shift by about 15 ppm on passing from the free aminoacids to their chelates with Pt(II), but only by about 3 ppm in the ternary complexes, thus confirming the non existence of the Pt-O bonds in the latter case.

$^{195}$Pt-NMR

The $^{195}$Pt-NMR spectra of the compounds *cis*-[$(NH_3)_2$Pt(gly)]$NO_3$, *cis*-[$(NH_3)_2$Pt(guo)(gly)]$NO_3$ and *cis*-[$(NH_3)_2$Pt(cyd)(gly)]$NO_3$ were recorded in $D_2O$ solutions. They all consist of a single band at -2128, -2560 and -2625 ppm respectively. The first corresponds to a $PtN_3O$ coordination [25]. The two others to a $PtN_4$ coordination as expected [26].

IR Spectra

IR assignments, whenever possible, were based on deuteration experiments and literature data [3,4,8,9]. Characteristic bands are included in Table III.

The region of 2800-3500 cm$^{-1}$ is covered by a very strong and broad band containing all the $\nu NH_2$, the $\nu$OH and aliphatic and aromatic $\nu$CH motions. This broad band shifts to about 2400 cm$^{-1}$ upon deuteration.

In the region however 1500-1700 cm$^{-1}$ the series of complexes *cis*-[$(NH_3)_2$Pt(guo)(amac)]$NO_3$ show a strong band near 1700 cm$^{-1}$, which is due to the overlapping of the $\nu$C=O of guo and of the $\nu^{as}(COO^-)$ of amac. This indicates $N_7$ coordination of guo and -$NH_2$ coordination of the aminoacids with the metal, as in other similar cases [3,4,27]. The other two maxima at 1635 and 1590 cm$^{-1}$ have been assigned to $\delta NH_2$ and purine skeletal vibrations.

In the series *cis*-[$(NH_3)_2$Pt(cyd)(amac)]$NO_3$ on the other hand, the strong band near 1650 cm$^{-1}$ has been attributed to the $\nu$C=O motion of cyd overlapped with the $\nu^{as}(COO^-)$ of the aminoacids. These indicate $N_3$ coordination of cyd and -$NH_2$ of the aminoacids [8,27].

Further in the guo ternary systems a decrease in the intensity of the band at 825 cm$^{-1}$ is observed, together with an increase of the one at 802 cm$^{-1}$, compared to the free guo. This indicates an increase of the percentage of the $C_{3'}$-*endo* ($^3E$) sugar conformation in the complexes, as it is also suggested from the $^1$H-NMR spectra (See *Sugar Conformation*).

### Ligand-Ligand Interactions

$^1$H-NMR spectra reveal the existence of hydrophobic ligand-ligand interactions in solution. Thus, the chemical shifts of the α -aliphatic protons of the aminoacids near the bonding sites with Pt(II), are observed upfield by about 0.7 ppm, on passing from the binary to the ternary complexes [8,18,19,28]. The shifts of the other aminoacid protons are also upfield in the ternary complexes, compared to the binary ones, but decreased with distance from the bonding sites (See Table I).

More particularly, the difference in chemical shifts, Δδ=δ amacH(free)-δ amac(tern.complex) between the zwitterionic forms of the aminoacids and their ternary complexes, given in Table IV, shows quantitatively the strength of ligand-ligand interactions [3,8,9]. They are larger in the case of guo complexes than cyd (See Figure 1).

Table V contains the difference in the chemical shifts of the terminal methyl groups of the anionic forms of the aminoacids and their ternary Pt(II) complexes, Δδ(ppm)=δamac-δtern.complex. The more positive Δδ values, the stronger the hydrophobic ligand-ligand interactions [8,28,29]. Thus,they increase with longer aliphatic side chain and they are stronger in the ternary guo complexes than in the cyd ones, as expected.

Figure I. (a) Difference in chemical shifts Δδ(ppm) of the protons of the zwitterionic form of nleu and the coordinated nleu in *cis*-$[(NH_3)_2Pt(guo)(nleu)]NO_3$ (◆) and *cis*-$[(NH_3)_2Pt(cyd)(nleu)]NO_3$ (■) ternary complexes (Positive Δδ values correspond to upfield shift upon coordination). (b) Variation of the Δδ(ppm) of the terminal methyl groups, of the anionic forms and the $-NH_2$ coordinated aminoacid anions in *cis*-$[(NH_3)_2Pt(guo)(amac)]NO_3$ (◆) and *cis*-$[(NH_3)_2Pt(cyd)(amac)]NO_3$ (■) ternary complexes.

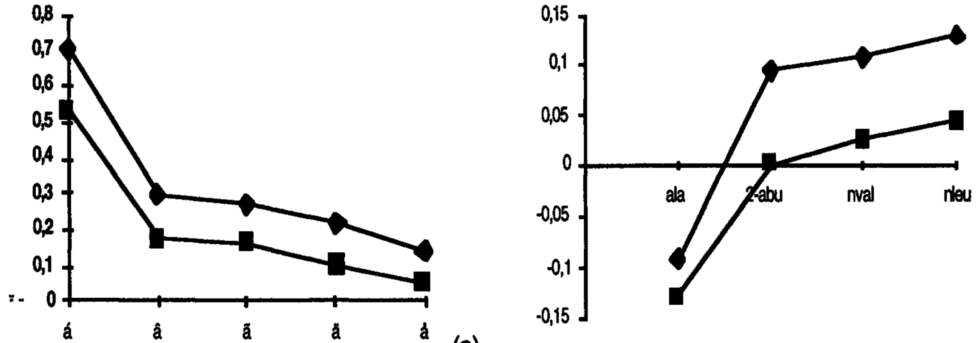

(a) (b)

Like in other similar cases [8,11], a hindered rotation around the $Pt-N_3$ bonding of the ternary complexes with cyd is also observed in the present system, except the complex containing also gly (See Figure II). Both $H_6$ and $H_5$ of cyd are shown up as two doublets in all the other cases containing chiral aminoacids [30-32] and it persists even at 90°C. Two sets of signals are also observed for the $H_{1'}$ sugar protons of cyd and two triplets for the á protons of the aminoacids (except glycine). This hindered rotation around the $Pt-N_3$ bonding was assigned to two diastereoisomers of head to tail oriented nucleobases and due to ortho substituents of cytidine [30,33].

### Sugar Conformation

Estimation of the $^3J_{HH}$ coupling constants from the $^1$H-NMR spectra and application of the Karplus equations [34-37] allows the calculation of the percentages of the various sugar conformations, i.e, $^3E$=$C_{3'}$-*endo,anti* and $^2E$=$C_{2'}$-*endo,anti* conformations of the furanose ring, the conformations around the $C_{4'}$-$C_{5'}$ bond of the sugar moiety, described also as *gg, gt* and *tg* conformations (See Figure III) and the *syn* and *anti* conformations of the sugar in the nucleosides.

The $^2E$, $^3E$ and *gg* % conformations can be calculated from the equations:

$$X_{2E} = \frac{J_{1'2'}}{J_{1'2'}+J_{3'4'}} \quad (3) \qquad , \quad X_{3E} = \frac{J_{3'4'}}{J_{1'2'}+J_{3'4'}} \quad (4)$$

and

$$X_{3E} = \frac{J_{3'4'}}{J_{1'2'}+J_{3'4'}} \quad (5) \quad , \quad \text{with} \quad \Sigma = J_{4'5'}+J_{4'5''} \quad (6)$$

Figure II. Splitting of $H_6$, $H_5$ and $H_{1'}$ protons of Cyd in the *cis*-[$(NH_3)_2$Pt(cyd)(nval)]$NO_3$ ternary complex in the $^1$H-NMR spectrum ($D_2O$, pD=5.5)

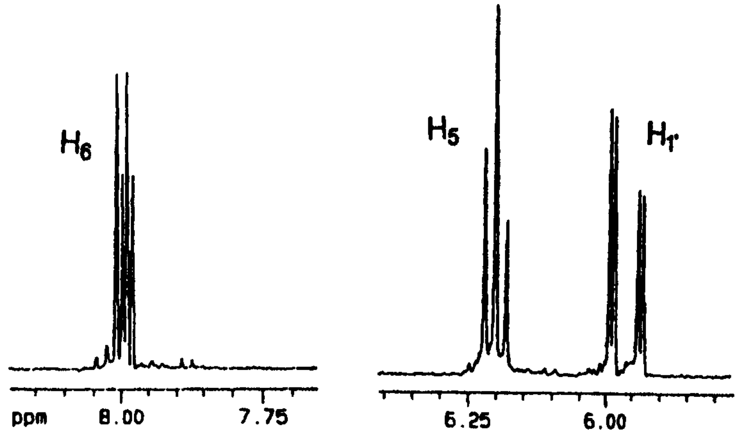

Figure III. (a) The $^3E$=$C_{3'}$-*endo,anti* and the $^2E$=$C_{2'}$-*endo,anti* conformations of the furanose ring. (b) The sugar *gg*, *gt* and *tg* conformations around the $C_{4'}$-$C_{5'}$ bond of the sugar moiety.

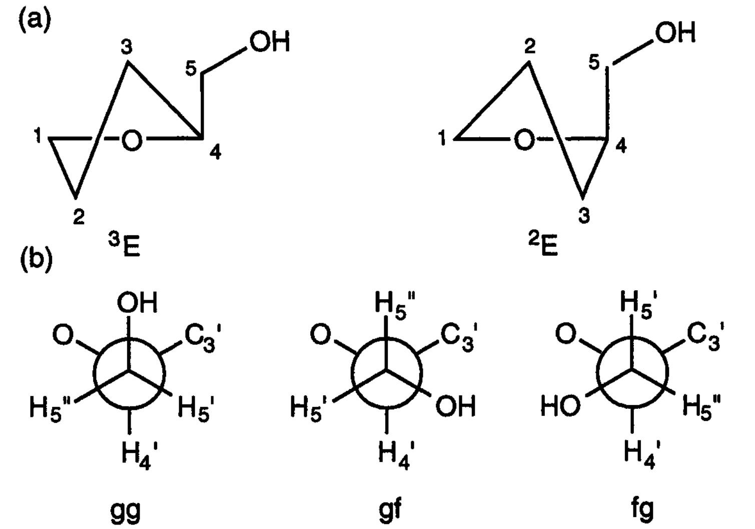

The $^1$H-NMR chemical shifts of the sugar protons, the coupling constants and the percentages of the various conformers are included in Table VI.

The percentage of the $^3E$ conformation increases by about 5-7% from the value of 38% for free guo [35], in the ternary complexes with guo and it does not depend on the aminoacid. An increase of about 8-11% was found also in the case of the complexes *cis*-[$(guo)_2$Pt(dipeptide ester)$_2$]$Cl_2$ [11] and 9-10% in *trans*-[$(guo)_2$Pt(amacH)$_2$]$Cl_2$ [7]. The increase was a little higher in the Pd(II) series of complexes, *cis*-[$(guo)_2$Pd(amac)]Cl [4] and *trans*-[$(guo)_2$Pd(dipeptide)$_2$]$Cl_2$ [10], 11-16%, but in no case it was depending on the aminoacid or the peptide.

The $^3E$ percentage in the ternary complexes with cyd however, does not appear to change from the value of the free ligand, ~60% [35].

The percentage of the *gg* conformation around the $C_{4'}$-$C_{5'}$ bond on the other hand, remains almost constant in the ternary complexes, compared to free guo, as it is also true when the ligand was coordinated to *cis*-DDP [38,39]. However, it was found to decrease slightly in other similar systems [2,7,11]. In the case of the ternary complexes with cyd on the other hand, it decreases by 4-9% (See Table 6).

The percentage of the *syn-anti* conformations can be calculated from the relationship $\delta_{obs} = P_{syn}\delta_{syn} + P_{anti}\delta_{anti}$ (7), with ä the chemical shift of the $H_{2'}$ proton, assuming 100% *syn* conformation for t-Buguo [39] with ä = 5.073 ppm and 100% *anti* conformation for the "closed" form of the compound *trans*-$[(guo)_2Pd(dipeptide)_2]Cl_2$ [3] with ä = 4.37 ppm. The results show a very slight increase of the *anti* conformation on passing from the free ligand to the ternary complexes. The increase of the *anti* conformation was much larger however in the *cis*-$[(guo)_2Pt(dipeptide\ ester)_2]Cl_2$ system [11].

*Conformation Around the $C_á$-$C_â$ Bond of the Aminoacids*

The percentage of the three possible conformations around the $C_á$-$C_â$ bond, *g, h, t,* designed in Figure 4 for aminoacids chelates, could be calculated in the case of the ternary complexes with cyd, where the sum of the coupling constants $J_{ÁÁ}+J_{BC}$ (Hz) [8,9,11,40] could also be calculated. In the case of the ternary complexes with guo, this sum could not be calculated, due to overlapping with sugar protons.

Figure IV. The three possible conformations *h, g, t* of the aminoacid chelates.

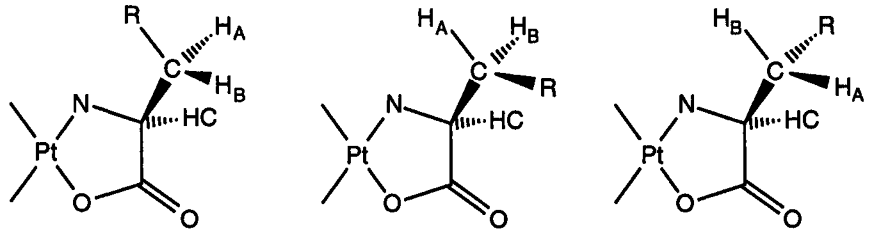

*h* *g* *t*

The results are included in Table VII.

Table VII: Vicinal coupling constants and rotamerdistribution in the ternary complexes with Cyd

| Compound | $J_{AB+BC}$ (Hz) | *h*(%) | *t+g*(%) | K |
|---|---|---|---|---|
| 2-abaH | 11.74 | 36.4 | 63.6 | 1.14 |
| *cis*-$[(NH_3)_2Pt(2\text{-}aba)]NO_3$* | 10.11 | 51.3 | 48.7 | 2.11 |
| *cis*-$[(NH_3)_2Pt(cyd)(2\text{-}aba)]NO_3$ | 12.29 | 31.3 | 68.7 | 0.91 |
| nvalH* | 12.24 | 31.8 | 68.2 | 0.93 |
| *cis*-$[(NH_3)_2Pt(nval)]NO_3$* | 10.02 | 52.1 | 47.9 | 2.18 |
| *cis*-$[(NH_3)_2Pt(cyd)(nval)]NO_3$ | 12.59 | 28.5 | 71.5 | 0.80 |
| nleuH | 11.64 | 37.2 | 62.8 | 1.18 |
| *cis*-$[(NH_3)_2Pt(nleu)]NO_3$ | 10.50 | 47.4 | 52.6 | 1.80 |
| *cis*-$[(NH_3)_2Pt(cyd)(nleu)]NO_3$ | 12.64 | 28.1 | 71.9 | 0.78 |

Taken from Ref. 14, **Calculated from K=h/(1-h)/2

As expected [8,9,11], the percentage of the *h* conformer which directs the aliphatic side chain towards the metal ion (Fig. IV), increases first on passing from the free aminoacids to their chelates with Pt(II), by about 10-20%. However, on passing to the ternary complexes, a decrease of about 19-24% of the *h* conformer is again observed, with values lower than the ones of the free ligands. This is possibly due to the monocoordination of the aminoacids with Pt(II) through the terminal $-NH_2$ group alone and to the ligand-ligand interactions taking place in the ternary complexes.

### References

1. Kasselouri, S.; Garoufis, A.; Hadjiliadis, N., *Inorg. Chim. Acta*, 1987, **135**, L23.
2. Garoufis, A.; Haran, R.; Pasdeloup, M.; Laussac, J. P.; Hadjiliadis, N., *J. Inorg. Biochem.*, 1987, **31**, 65.
3. Kasselouri, S.; Laussac, J. P.; Hadjiliadis, N., *Inorg. Chim. Acta*, 1989, **166**, 239.
4. Kasselouri, S.; Hadjiliadis, N., *Inorg. Chim. Acta*, 1990, **168**, 15.
5. Schwarz, F.; Lippert, B.; Iakovidis, A.; Hadjiliadis, N., *Inorg. Chim. Acta*, 1990, **168**, 275.

6. Iakovidis, A.; Hadjiliadis, N.; Dahan, F.; Laussac, J. P.; Lippert, B., *Inorg. Chim. Acta,* 1990, **175**, 57.
7. Garoufis, A.; Hatiris, J.; Hadjiladis, N., *J. Inorg. Biochem.*, 1991, **41**, 195.
8. Iakovidis, A.; Hadjiliadis, N.; Britten, I. E.; Butler, I. S.; Schwarz, F.; Lippert B., *Inorg. Chim. Acta,* 1991, **184**, 209.
9. Aletras, V.; Hadjiliadis, N.; Lippert, B., *Polyedron,* 1992, **11**, 1359.
10. Aletras, V.; Hadjiliadis, N.; Lymberopoulou-Karaliota, A.; Rombeck, I.; Lippert, B., *Inorg. Chim. Acta,* 1994, **227**, 17.
11. Katsarou, E.; Troganis, A.; Hadjiliadis, N., *Inorg. Chim. Acta,* in press.
12. Dhara S. G., *Indian. J. Chem.*, 1970, **8**, 193.
13. Boreham, C. J.; Broomhead, J. A.; Fairlie, D. P., *Aust. J. Chem.*, 1981, **34**, 659.
14. Iakovidis, A.; Hadjiliadis, N.; Schollhorn, H.; Thewalt, U.; Trotscher, G., *Inorg. Chim. Acta,* 1989, **164**, 221.
15. Pivcova, H.; Sandek, V.; Noskova, D.; Drobnik, J., *J. Inorg. Biochem.*, 1985, **23**, 43.
16. Hadjiliadis, N.; Pneumatikakis, G., *Inorg. Chim. Acta,* 1980, **46**, 225.
17. Kong, P. C.; Theophanides, T., *Inorg. Chem.*, 1974, **13**, 1167.
18. Hadjiliadis, N.; Theophanides, T., *Inorg. Chim. Acta,* 1976, **16**, 74.
19. Polissiou, M.; Phan Viet, M. T.; St Jaques, M.; Theophanides, T., *Inorg. Chim. Acta,* 1985, **107**, 203..
20. Marzilli, L. G.; de Castro, B.; Solorzano, C., *J. Am. Chem. Soc.*, 1982, **104**, 461.
21. Marzilli, L. G.; Cholilpoyil, P., *J. Am. Chem. Soc.*, 1980, **102**, 873.
22. Barbarella, G.; Bertoluzzo, A.; Morelli, M. A.; Tosi, M. R.; Tugnoli, V., *Gaz. Chim. Ital.*, 1988, **118**, 637.
23. Hadjiliadis, N; Pneumatikakis, G., *Inorg. Chim. Acta,* 1980, **46**, 255.
24. Trovo, G.; Valle, G.; Longato, B., *J. Chem. Soc. Dalton,* 1993, 669.
25. Appleton, T. G.; Halland, J. R.; Ralph, S. F., *Inorg. Chem.*, 1985, **24**, 673.
26. Pregosin, P. S., Coord. Chem. Revs., 1982, **44**, 247.
27. Mathlonthi, M.; Seuvre A.; Koenig, J. L., *Carbohydrate Res.*, 1986, **146**, 15.
28. Fisher, B. E.; Sigel, H., *J. Am. Chem. Soc.*, 1980, **102**, 2998.
29. Sigel. H.; Fischer, B. E.; Farkas, E., *Inorg. Chem.*, 1983, **22**, 925.
30. Reily, D. M.; Wilkowski, K.; Shinozuka, K.; Marzilli, L. G., *Inorg. Chem.*, 1985, **24**, 37.
31. Reily, D. M.; Marzilli, L.G., *J. Am. Chem. Soc.*, 1986, **108**, 6785.
32. Inagaki, K.; Dijt, F.J.; Lempers, E. L. M.; Reedijk, J., *Inorg. Chem.*, 1988, **27**, 382.
33. Song, B.; Feldmann, G.; Bastian, M.; Lippert, B.; Sigel, H., *Inorg. Chim. Acta,* 1995, **235**, 99.
34. Lee, C. H.; Sarma, R. H., *J. Am. Chem. Soc.*, 1976, **98**, 3541.
35. Kim, S. H.; Sarma, R. H., *J. Am. Chem. Soc.*, 1978, **100**, 1571.
36. Lee, C. H.; Esra, F. S.; Kondo, N. S.; Sarma, R. H.; Danyloux, S. S., *Biochemistry,* 1976, **15**, 3627.
37. Esra, F. S.; Lee, C. H.; Kondo, N.S.; Danyloux, S. S.; Sarma, R. H., *Biochemistry,* 1977, **16**, 1977.
38. Okamoto, K.; Benhau, V.; Gauthier, J. V.; Hanessian, S.; Theophanides, T., *Inorg. Chim. Acta,* 1986, **123**, L1.
39. Polissiou, M.; Theophanides, T., *Inorg. Chim. Acta,* 1987, **137**, 195.
40. Kim, S. H.; Martin, R. B., *J. Am. Chem. Soc.*, 1984, **106**, 1707.
41. Altona, C.; Sundaralingam, M., *J. Am. Chem. Soc.*, 1972, **94**, 2333.

**Received: February 3, 1997 - Accepted: March 5, 1997 - Received in revised camera-ready format: March 5, 1997**

